# Effectiveness and Safety of Fixed-Dose Empagliflozin/Linagliptin Combination in Type 2 Diabetes Mellitus: Real-World Evidence From Bangladesh

**DOI:** 10.7759/cureus.102858

**Published:** 2026-02-02

**Authors:** Indrajit Prasad, Ajit K Paul, M. Saifuddin, Nusrat Sultana, Mashfiqul Hasan, Masud-Un Nabi, AKM A Islam, Moinul Islam, Mohammad Atiqur-Rahman, Mohammad I Mahbub

**Affiliations:** 1 Department of Endocrinology, Dhaka Medical College Hospital, Dhaka, BGD; 2 Department of Endocrinology, Mainamoti Medical College and Hospital, Comilla, BGD; 3 Department of Endocrinology, Bangladesh Medical University, Dhaka, BGD; 4 Department of Endocrinology, Rajshahi Medical College, Rajshahi, BGD; 5 Department of Endocrinology, Sir Salimullah Medical College and Mitford Hospital, Dhaka, BGD; 6 Department of Endocrinology, National Institute of Neurosciences and Hospital, Dhaka, BGD; 7 Department of Endocrinology, National Institute of Burn and Plastic Surgery, Dhaka, BGD

**Keywords:** bangladesh, cardiometabolic benefits, empagliflozin-linagliptin, fixed-dose combination, glycemic control, type 2 diabetes mellitus

## Abstract

Background: Type 2 diabetes mellitus (T2DM) presents a growing health burden globally and in Bangladesh, where control rates remain suboptimal. Fixed-dose combinations (FDCs) integrating agents with complementary mechanisms, such as empagliflozin and linagliptin, may enhance glycemic control, reduce cardiovascular risk factors, and improve adherence. This study aimed to evaluate the real-world effectiveness and safety of empagliflozin-linagliptin FDC over 24 weeks in adult T2DM patients in Bangladesh.

Methods: This prospective, multi-center, open-label, real-world observational cohort study was conducted across 10 outpatient centers in routine clinical practice in Bangladesh and enrolled 321 adults with T2DM who were either treatment-naïve or inadequately controlled on oral antidiabetic drugs. Participants received once daily empagliflozin 10 mg or 25 mg, plus linagliptin 5 mg. Clinical visits were conducted at baseline, week 6, week 12, and week 24. Primary endpoints comprised changes in HbA1c and fasting plasma glucose (FPG). Secondary measures included proportions achieving HbA1c <7%, and changes in weight, blood pressure, lipid profile, renal function, and liver enzymes. Safety was assessed via recorded adverse events and treatment discontinuation rates.

Results: At 24 weeks, mean HbA1c decreased significantly from 9.82 ± 1.01% to 6.29 ± 0.76% (mean change: -3.55%, n = 303, p<0.001). FPG dropped from 13.27 ± 2.66 mmol/L to 6.31 ± 0.70 mmol/L (mean change: -6.96 mmol/L, n = 321, p < 0.001), and 225 (74.3%) attained HbA1c <7%. There were significant reductions in mean weight (-7.17 kg), systolic blood pressure (-14.97 mmHg), and diastolic blood pressure (-2.84 mmHg) (n = 321, p < 0.001 for all). Among subsets, total cholesterol, low-density lipoprotein (LDL), triglycerides, serum creatinine, and serum glutamic pyruvic transaminase (SGPT) levels improved, with estimated glomerular filtration rate (eGFR) increasing significantly (p < 0.05). No serious adverse events or study withdrawals occurred; minor adverse events (AEs) included transient hypoglycemia in one participant (<1%), anorexia or nausea in 10 (≤3.1%), urinary tract infections in two (~0.6%), dysuria in two (<1%), and dizziness in two (<1%).

Conclusion: In this real-world observational cohort of Bangladeshi adults with T2DM, treatment with the empagliflozin-linagliptin FDC was associated with improvements in glycemic control, cardiometabolic benefits, and renal improvements over 24 weeks, with a favorable short-term safety profile. These findings indicate that the FDC was associated with significant improvements in this real-world cohort, providing supportive evidence for its utility.

## Introduction

Type 2 diabetes mellitus (T2DM) is a chronic metabolic disorder characterized by impaired insulin secretion and insulin resistance, leading to persistent hyperglycemia [1]. Over the past few decades, the global prevalence of T2DM has risen steadily, becoming a significant public health concern [2]. Its global prevalence has risen markedly, making T2DM a major public health concern. According to the International Diabetes Federation (IDF), diabetes affects 10.5% of adults worldwide, with nearly 45% of cases remaining undiagnosed, and the number of affected individuals is projected to reach 783 million by 2045 [3]. The burden is disproportionately higher in low- and middle-income countries, where the majority of diabetes-related morbidity, mortality, and healthcare expenditure occurs [4].

In the IDF South-East Asia region, 90 million adults were living with diabetes in 2021, a number expected to increase substantially by 2045 [3]. In Bangladesh, diabetes prevalence among adults is estimated at 12.5%, affecting more than 13 million individuals, with a large proportion remaining undiagnosed or inadequately treated [3-5].

T2DM exhibits insulin resistance in muscle, liver, and adipose tissue, with a progressive deterioration of pancreatic beta cell function [5]. Given the complex pathophysiology of T2DM, an ideal combination of oral antidiabetic drugs (OADs) should offer complementary mechanisms of action and provide durable glycemic control. Additionally, these drugs should exhibit good tolerability with minimal risk of hypoglycemia or weight gain and potentially provide cardiovascular benefits [6,7]. The recommended guideline for the treatment of T2DM is to initiate OAD when diet and exercise are inadequate. Regardless of the first-line OAD, the majority of patients eventually require a second drug with a complementary mechanism to maintain glycemic control [8].

Sodium-glucose co-transporter 2 (SGLT2) inhibitors have emerged as effective add-on therapies at various stages of diabetes, especially in patients with a high risk of atherosclerotic cardiovascular disease (ASCVD) or weight concerns [7]. Empagliflozin is a specific SGLT2 inhibitor that works by reducing renal glucose reabsorption in the proximal tubules and increasing urinary glucose excretion (glycosuria), thereby lowering blood glucose levels in individuals with T2DM [9]. It should be noted that the cardiovascular and renal benefits associated with SGLT2 inhibitor therapy have been most consistently demonstrated in patients with established cardiovascular disease, heart failure, or chronic kidney disease. Besides improving glycemic control (as measured by HbA1c and fasting plasma glucose (FPG)), SGLT2 inhibitors help lower body weight, systolic blood pressure (SBP), and cardiovascular mortality in high-risk individuals [10]. Recent trials have also shown that SGLT2 inhibitors reduce hospitalization for heart failure in patients with T2DM, many of whom had coexisting ASCVD [11]. Empagliflozin was approved in the United States in 2014 as an adjunct to diet and exercise for glycemic control in adults with T2DM. It is available in 10 mg and 25 mg doses, taken once daily with or without food [12].

Dipeptidyl peptidase-4 (DPP-4) inhibitors, such as linagliptin, enhance incretin activity by preventing the degradation of peptides like GLP-1. DPP-4 inhibitors have demonstrated a neutral cardiovascular safety profile in large outcome trials [13], and when combined with SGLT2 inhibitors, agents with established cardiovascular and renal benefits [10], provide complementary glucose-lowering without offsetting cardioprotective effects. This leads to glucose-dependent insulin secretion and suppression of glucagon, resulting in reduced blood glucose levels [8]. A single oral dose of 5 mg linagliptin inhibits approximately 90% of DPP-4 activity, with the effect sustained for up to 24 hours. Linagliptin is primarily eliminated via the hepatobiliary route, making it suitable for use across all levels of renal function without dose adjustment [14]. It was first approved in the US in 2011 and is widely used at a standard dose of 5 mg once daily [12].

Both SGLT2 inhibitors and DPP-4 inhibitors carry a low risk of hypoglycemia, as SGLT2 inhibitors work via insulin-independent pathways, and DPP-4 inhibitors promote glucose-dependent insulin secretion [8]. However, due to the chronic and progressive nature of T2DM, maintaining glycemic targets with monotherapy becomes increasingly difficult over time. Combination therapy is often necessary but may lead to treatment fatigue due to pill burden and adherence challenges. Fixed-dose combinations (FDCs) have been shown to reduce pill burden and may improve medication adherence in real-world settings, which is particularly relevant for chronic conditions such as T2DM [15].

FDCs of empagliflozin and linagliptin have shown promise in patients inadequately controlled on either empagliflozin plus metformin or linagliptin plus metformin. These combinations offer dual benefits: glycemic control and cardiovascular-renal protection [16]. Large clinical trials, including EMPA-REG OUTCOME, EMPEROR-Reduced, and CARMELINA, have demonstrated the cardiovascular and renal benefits of empagliflozin and linagliptin in high-risk T2DM patients as individual agents. It should be noted that these trials primarily evaluated the separate components rather than the empagliflozin-linagliptin FDC itself. The empagliflozin/linagliptin FDC was approved by the US FDA on January 30, 2015 [17], by the European Union in November 2016 [18], and by the Directorate General of Drug Administration (DGDA) in Bangladesh on April 10, 2022.

Despite these developments, long-term clinical experience with these drug classes remains limited, particularly concerning safety and tolerability in local populations. Given the scarcity of local data, the current study has been structured as a real-world, observational cohort study to assess the efficacy and safety of empagliflozin-linagliptin FDC therapy in routine clinical practice in Bangladesh. Therefore, the present study aims to assess the real-world outcomes of empagliflozin and linagliptin FDC therapy in adults with T2DM in Bangladesh.

## Materials and methods

Study design and participants

This study was a prospective, multi-center, open-label observational cohort design conducted at 10 outpatient facilities within the framework of routine clinical practice in Bangladesh. The study included male and female patients diagnosed with T2DM, aged 18 to 65 years. Participants were either treatment-naïve or inadequately controlled, defined by baseline HbA1c levels exceeding 8% but not surpassing 10.5%, and/or FPG levels ranging from 7 to 15 mmol/L. These criteria were established in alignment with the guidelines set forth by the Diabetic Association of Bangladesh (BADAS) [19]. Outcomes were analyzed across the overall cohort and were not stratified by prior treatment status. Exclusion criteria included individuals with any form of diabetes other than T2DM, patients currently receiving insulin therapy, those with a history of recurrent genitourinary infections, severe liver damage, or significant renal impairment (estimated glomerular filtration rate (eGFR) <25 mL/min/1.73 m^2^). Furthermore, individuals who were critically ill, mentally unstable, substance abusers, cancer patients, pregnant or lactating women, or those with any other condition that the attending physician deemed a contraindication to participation were also excluded from the study. The study was conducted from August 2023 to July 2024 at the outpatient departments of multiple centers across Bangladesh.

Using real-world data from a one-year, multicenter post-marketing surveillance study of the empagliflozin/linagliptin FDC in Japanese adults with type 2 diabetes, where the 24-week mean change in HbA1c was -0.39% with a standard deviation (SD) of 1.11 [20], we recalculated based on a single-arm mean change framework. Assuming a one-sided significance level (α) of 0.05 and 90% power, the minimum required sample size was estimated to be 171 participants. To preserve power under an anticipated attrition of 40%, the target enrollment was inflated to 286 participants. These assumptions, along with the underlying dispersion and effect estimates, are consistent with the contemporary real-world performance of the FDC.

Study procedure

The participants were started on once-daily therapy with empagliflozin 10 mg plus linagliptin 5 mg or empagliflozin 25 mg plus linagliptin 5 mg. The determination of the empagliflozin dosage (10 mg or 25 mg, administered once daily) was at the discretion of the treating physician, consistent with standard clinical practice. Dose selection was personalized based on the physician's clinical judgment, which considered factors such as the patient's baseline glycemic control, existing comorbid conditions, and perceived tolerability of the medication. There were no established, protocol-driven criteria for allocating doses. For each, concomitant treatments for dyslipidemia, hypertension, and other associated diseases were continued in accordance with routine clinical care. Information on concomitant medications was recorded at each study visit; however, no protocol-mandated restrictions or adjustments were applied. Study visits were scheduled at baseline (Visit 1) and subsequently at six weeks (Visit 2), 12 weeks (Visit 3), and 24 weeks (Visit 4) of treatment. Each visit was permitted to occur within a window of ±1 week from the designated target date.

During Visit 1, socio-demographic and clinical data were collected. Physical measurements, including height, weight, and blood pressure, were recorded. A detailed medical history documenting conditions such as hypertension, ischemic heart disease, hyperlipidemia, hypothyroidism, and chronic kidney disease was also obtained. Additionally, laboratory test results, including FPG, HbA1c (%), serum glutamic-pyruvic transaminase (SGPT), serum creatinine/eGFR, lipid profile, and urine routine examination, were performed at local laboratories of the participating centers, in accordance with routine clinical practice. Centralized laboratory testing was not employed due to the observational, real-world nature of the study.

In the subsequent visits, specific data points were systematically monitored. At all follow-up visits, weight and blood pressure measurements were documented. Adverse events were recorded, along with the details of any concomitant medications. Adverse events were actively monitored at each study visit through direct patient interviews. Reports from urine routine examinations were also obtained at each visit. Additionally, during Visit 2, FPG reports were collected, while Visit 3 included both FPG and HbA1c reports. Analyses of renal function, lipid profile, and liver enzyme parameters were conducted in participants with available laboratory data at baseline and week 24. These assessments were part of routine clinical care and were not uniformly available for all enrolled patients. Accordingly, these outcomes were analyzed as secondary and exploratory endpoints.

Outcome measures

The primary efficacy outcomes of the study included the change from baseline in HbA1c and FPG levels at week 24. The secondary efficacy outcomes were the percentage of patients who achieved the target HbA1c level of less than 7% after 24 weeks, as well as the mean changes in SBP, diastolic blood pressure (DBP), and body weight from baseline to week 24. Body mass index (BMI) was calculated as weight in kilograms divided by height in meters squared (kg/m^2^). Participants were categorized as healthy weight (18.5 to <25.0 kg/m^2^), overweight (25.0 to <30.0 kg/m^2^), and obese (≥30.0 kg/m^2^). The primary safety outcome of the study was the percentage of patients who withdrew from the study due to adverse events. Secondary safety outcomes were the total number of adverse events and serious adverse events reported. Adverse events of special interest included confirmed hypoglycemic events (defined as plasma glucose levels <3.9 mmol/L and/or requiring assistance), diabetic ketoacidosis, pancreatitis, events consistent with urinary tract infections (UTIs), genital infections, and hypersensitivity reactions. Adverse events of special interest were passively reported during the study, based on spontaneous reporting by investigators and participants, consistent with routine clinical practice. No additional active surveillance procedures were implemented.

Statistical analysis

All data were expressed as mean ± SD for continuous variables and as frequencies with percentages for categorical variables. Parametric statistical methods were used to analyze continuous variables. Formal tests of normality were not performed, and non-parametric methods were not used. Changes in HbA1c, FPG, blood pressure, body weight, lipid profiles, SGPT, and eGFR from baseline to week 24 were analyzed using paired t-tests. Analyses were performed using available data at each time point without imputation for missing values; therefore, the number of patients included in analyses may vary across visits. No formal adjustment for multiple comparisons was applied. All statistical analyses were conducted using IBM SPSS Statistics for Windows, Version 25 (Released 2017; IBM Corp., Armonk, New York, United States), and p-values < 0.05 were considered statistically significant.

## Results

A total of 321 patients diagnosed with T2DM participated in this study. Most participants were aged between 46 and 59 years, comprising 141 (43.9%) of the cohort, and 197 (61.4%) were female. Individuals classified as overweight represented 147 (45.8%) of the population, while 131 (40.8%) had been living with diabetes for over a decade. The prevalence of common comorbidities included hypertension at 104 (32.4%), hyperlipidemia at 72 (22.4%), and ischemic heart disease at 50 (15.6%) (Table 1).

**Table 1:** Baseline characteristics of the study participants BMI: body mass index; HTN: hypertension; IHD: ischemic heart disease; CKD: chronic kidney disease; MASLD: metabolic dysfunction-associated steatotic liver disease

Variable	Frequency	Percentage (%)
Age		
≤45 years	95	29.6
46-59 years	141	43.9
≥60 years	85	26.5
Gender		
Male	124	38.6
Female	197	61.4
BMI		
Healthy	89	27.7
Overweight	147	45.8
Obesity	85	26.5
Duration of diabetes		
<5 years	72	22.4
6-10 years	118	36.8
>10 years	131	40.8
Comorbidities		
HTN	104	32.4
IHD	50	15.6
Hyperlipidemia	72	22.4
Hypothyroidism	22	6.9
CKD	46	14.3
MASLD	9	2.8

**Figure 1:**
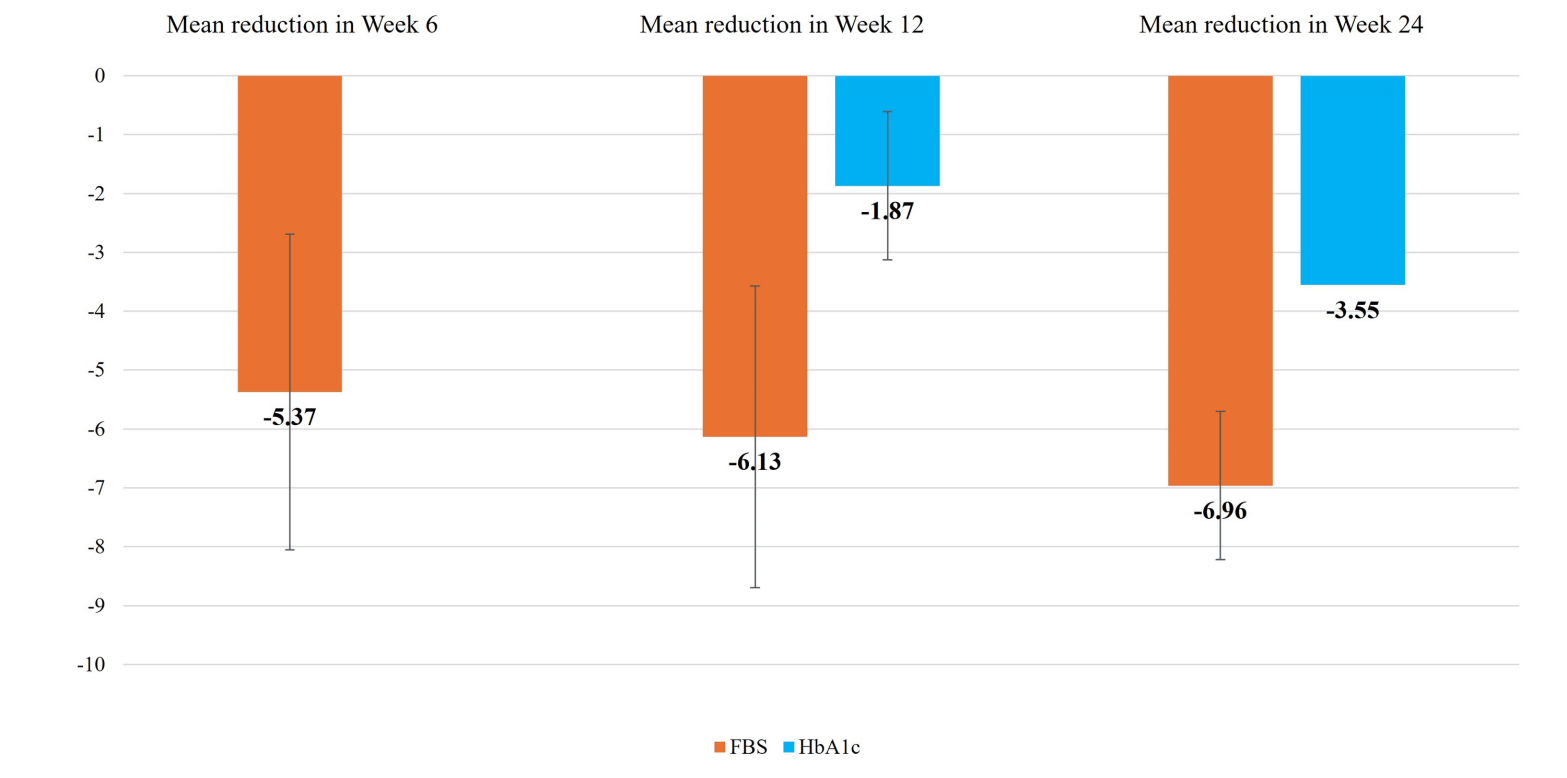
Mean reduction in HbA1c (%) and fasting plasma glucose (FPG, mmol/L) among study participants HbA1c: glycated hemoglobin; FBS: fasting blood sugar

Table 2 presents the efficacy outcomes of the study participants. The mean HbA1c significantly decreased from 9.82 ± 1.01% at baseline to 7.97 ± 1.68% at 12 weeks and further to 6.29 ± 0.76% at 24 weeks, with respective mean reductions of 1.87% and 3.55% from baseline (n = 303, p < 0.001 for both). Similarly, the mean FPG decreased significantly from 13.27 ± 2.66 mmol/L at baseline to 7.93 ± 1.14 mmol/L at six weeks, 7.14 ± 0.90 mmol/L at 12 weeks, and 6.31 ± 0.70 mmol/L at 24 weeks, with respective reductions of 5.37, 6.13, and 6.96 mmol/L (n = 321, p < 0.001 for all comparisons) (Figure 1). In addition, 225 (74.25%) participants successfully achieved an HbA1c level below 7%. From baseline, mean weight decreased by 7.17 ± 4.41 Kg (n = 321, p < 0.001), SBP by 14.97 ± 14.35 mm of Hg (n = 321, p < 0.001), and DBP by 2.84 ± 9.0 mm of Hg (n = 321, p < 0.001) by the 24-week mark.

**Table 2:** Change of efficacy outcome variables of the study participants HbA1c: glycated hemoglobin; FPG: fasting plasma glucose; SBP: systolic blood pressure; DBP: diastolic blood pressure

Variables	Mean±SD	Change from baseline	P-value (two-tailed)
HbA1c (%)			
Visit 1 (Baseline)	9.82±1.01	-	-
Visit 3 (12 Weeks)	7.97±1.68	1.87±1.63	<0.001
Visit 4 (24 Weeks)	6.29±0.76	3.55±1.26	<0.001
FPG (mmol/L)			
Visit 1 (Baseline)	13.27±2.66	-	-
Visit 2 (6 Weeks)	7.93±1.14	5.37±2.68	0.001
Visit 3 (12 Weeks)	7.14±0.90	6.13±2.56	<0.001
Visit 4 (24 Weeks)	6.31±0.70	6.96±1.26	<0.001
Weight (Kg)			
Visit 1 (Baseline)	69.76±11.97	-	-
Visit 2 (6 Weeks)	67.50±12.19	2.30±3.52	<0.001
Visit 3 (12 Weeks)	65.15±11.79	4.53±3.97	<0.001
Visit 4 (24 Weeks)	62.59±11.71	7.17±4.41	<0.001
SBP (mm of Hg)			
Visit 1 (Baseline)	133.82±13.98	-	-
Visit 2 (6 Weeks)	121.95±5.72	12.05±12.88	<0.001
Visit 4 (24 Weeks)	119.12±4.60	14.97±14.35	<0.001
DBP (mm of Hg)			
Visit 1 (Baseline)	81.35±6.61	-	-
Visit 2 (6 Weeks)	80.17±5.28	1.14±7.98	0.012
Visit 4 (24 Weeks)	78.48±6.07	2.84±9.0	<0.001

The study also investigated changes in lipid levels, renal function, and liver enzyme levels among participants from Visit 1 (baseline) to Visit 4 (24 weeks). Statistically significant improvements were observed across several parameters. Serum creatinine decreased from 1.14 ± 0.38 mg/dL to 0.89 ± 0.16 mg/dL (n = 95, p < 0.001), while eGFR significantly increased from 79.6 ± 27.41 mL/min/1.73m^2^ to 97.71 ± 17.54 mL/min/1.73m^2^ (n = 95, p < 0.001). For lipid profiles (n = 106), total cholesterol decreased from 240.46 ± 49.07 mg/dL to 186.29 ± 20.58 mg/dL (p < 0.001), and low-density lipoprotein (LDL) decreased from 125.17 ± 32.50 mg/dL to 95.82 ± 16.85 mg/dL (p < 0.001). High-density lipoprotein (HDL) showed a slight but significant increase from 37.15 ± 3.91 mg/dL to 38.87 ± 5.71 mg/dL (p < 0.001), and triglycerides (TG) decreased from 185.86 ± 47.42 mg/dL to 155.82 ± 98.53 mg/dL (p = 0.011). Furthermore, serum glutamic pyruvic transaminase (SGPT) levels (n = 87) showed a significant reduction from 44.5 ± 22.25 IU/L to 40.28 ± 10.73 IU/L (p = 0.030) (Table 3).

**Table 3:** Change of lipid levels, renal function, and liver enzyme levels in the study participants eGFR: estimated glomerular filtration rate; LDL: low-density lipoprotein; HDL: high-density lipoprotein; TG: triglycerides; SGPT: serum glutamic pyruvic transaminase

Investigations	Visit 1	Visit 4	p-value
Serum creatinine (mg/dL)	1.14±0.38	0.89±0.16	<0.001
eGFR (mL/min/1.73m^2^)	79.6±27.41	97.71±17.54	<0.001
Cholesterol (mg/dL)	240.46±49.07	186.29±20.58	<0.001
LDL (mg/dL)	125.17±32.50	95.82±16.85	<0.001
HDL (mg/dL)	37.15±3.91	38.87±5.71	<0.001
TG (mg/dL)	185.86±47.42	155.82±98.53	0.011
SGPT (IU/L)	44.5±22.25	40.28±10.73	0.030

No patients withdrew from the study due to adverse events, and there was no incidence of serious adverse events. The overall incidence of adverse events was highest at Visit 2 (six weeks) with 16 reported cases (5.1%), predominantly anorexia/nausea (10 cases, 3.1%). By Visit 3 (12 weeks) and Visit 4 (24 weeks), the total adverse events decreased to six cases (1.9%) for both time points, with anorexia and nausea, as well as UTIs, being the most consistently reported events, albeit at low frequencies. Hypoglycemia occurred in single instances at six and 12 weeks. Other events, like dizziness and dysuria, were observed only at six weeks (Table 4).

**Table 4:** Adverse events of the study participants UTIs: urinary tract infections

Variables	Visit 2 (six weeks)	Visit 3 (12 weeks)	Visit 4 (24 weeks)
Hypoglycemia	1 (0.3%)	1 (0.3%)	0 (0%)
Anorexia/nausea	10 (3.1%)	3 (0.9%)	4 (1.2%)
Dizziness	2 (0.6%)	0 (0%)	0 (0%)
Dysuria	2 (0.6%)	0 (0%)	0 (0%)
UTIs	2 (0.6%)	2 (0.6%)	2 (0.6%)
Others	2 (0.6%)	0 (0%)	1 (0.3%)
Total	16 (5.1%)	6 (1.9%)	6 (1.9%)

## Discussion

In this real-world observational cohort of Bangladeshi adults with T2DM, treatment with the empagliflozin-linagliptin FDC was associated with improved glycemic control in routine clinical practice. These findings should be interpreted in the context of the high baseline glycemic burden and the non-randomized, observational study design. Favorable trends were also observed in body weight, blood pressure, and selected metabolic parameters; however, analyses of renal, lipid, and liver outcomes were based on incomplete laboratory data and should therefore be considered exploratory. No serious adverse events or treatment discontinuations were reported during follow-up.

The observed findings are broadly consistent with evidence from randomized controlled trials and real-world studies evaluating empagliflozin-linagliptin combination therapy across different populations. Randomized trials conducted in East Asian populations have demonstrated effective glycemic control with this FDC, along with favorable effects on body weight and blood pressure and good overall tolerability [18,20]. In addition, meta-analyses of SGLT2 inhibitor and DPP-4 inhibitor combination therapy have reported additive glycemic benefits with modest weight and blood pressure reductions [21]. Real-world studies from South Asia similarly suggest that FDCs may support glycemic improvement and treatment adherence in routine clinical settings [22]. Collectively, these data suggest a broadly consistent pharmacodynamic profile of empagliflozin-linagliptin therapy across diverse populations.

The magnitude of glycemic improvement observed in the present study should be interpreted cautiously. Patients with higher baseline HbA1c levels are more likely to experience larger absolute reductions following treatment initiation, a pattern that is clinically relevant but also partly attributable to regression to the mean. In the absence of a comparator group or formal analyses examining the relationship between baseline glycemic severity and the magnitude of change, it is not possible to fully disentangle treatment-associated effects from this statistical phenomenon. Consequently, the observed reductions should be viewed as real-world associations rather than definitive evidence of causal efficacy across the full spectrum of disease severity.

Several methodological considerations further warrant cautious interpretation. Changes in concomitant medications during follow-up were permitted as part of routine clinical care but were neither systematically captured nor adjusted for, potentially introducing confounding. Empagliflozin dose selection was based on physician discretion rather than prespecified criteria, reflecting real-world practice but limiting reproducibility and dose-specific inference. In addition, multiple paired comparisons were performed without formal multiplicity adjustment, increasing the risk of type I error, particularly for secondary outcomes such as lipid parameters and liver enzymes.

The study has several strengths, including its multicenter, real-world design, its relatively large overall cohort, and its assessment of multiple clinically relevant outcomes in routine practice. However, important limitations must be acknowledged. The observational, non-randomized design and absence of a control group limit causal inference. Laboratory outcomes were available only for subsets of participants, raising the possibility of selection bias and limiting the generalizability of secondary findings. The predominance of female participants may further restrict applicability to broader type 2 diabetes populations. Additionally, renal outcomes were not evaluated stratified by baseline kidney function, which limits interpretation across different stages of chronic kidney disease. Formal assessment of data normality and the use of non-parametric statistical methods were not undertaken. Although parametric tests are generally robust to moderate departures from normality, this may represent a methodological limitation and should be considered when interpreting the findings.

Taken together, these findings suggest that empagliflozin-linagliptin FDC therapy is associated with improved glycemic control and favorable cardiometabolic trends in routine clinical practice, while highlighting the need for cautious interpretation. Future studies employing randomized controlled designs, standardized dosing criteria, longer follow-up, and comprehensive adjustment for confounding factors are needed to confirm these observations and to better define the clinical benefits of this combination therapy across diverse patient subgroups.

## Conclusions

This real-world observational study in a Bangladeshi population with T2DM found that treatment with the empagliflozin-linagliptin FDC was associated with significant improvements in glycemic parameters, body weight, blood pressure, and selected renal and lipid markers over a 24-week period, with a favorable short-term safety and tolerability profile. Longer-term, controlled studies incorporating comparative arms, adjustment for confounders, and patient-centered outcomes are warranted to further define the role of this FDC across diverse populations.
